# Urban mental health: a position paper of the European psychiatric association

**DOI:** 10.1192/j.eurpsy.2025.10100

**Published:** 2025-09-03

**Authors:** Błażej Misiak, Julia Karska, Szymon Kowalski, Philippe Courtet, Umberto Volpe, Meryam Schouler-Ocak, Marianne Destoop, Kristina Adorjan, Fabian Kraxner, Victor J.A. Buwalda, Jonathan Campion, Julian Beezhold, Peter Falkai, Geert Dom

**Affiliations:** 1 Department of Psychiatry, Wroclaw Medical University, Wroclaw, Poland; 2 IGF, University of Montpellier, CNRS, INSERM, Montpellier, France; 3 Department of Emergency Psychiatry and Acute Care, Lapeyronie Hospital, CHU Montpellier, Montpellier, France; 4 Unit of Clinical Psychiatry, Department of Clinical Neurosciences/DIMSC, School of Medicine, Università Politecnica delle Marche, Ancona, Italy; 5 Psychiatric University Clinic of Charité at St. Hedwig Hospital, Berlin, Germany; 6 Multiversum Psychiatric Hospital, Brothers of Charity Belgium, Boechout, Belgium; 7 Collaborative Antwerp Psychiatry Research Institute (CAPRI), University of Antwerp, Antwerp, Belgium; 8 Institute of Psychiatric Phenomics and Genomics (IPPG), LMU University Hospital, LMU Munich, Munich, Germany; 9 University Hospital of Psychiatry and Psychotherapy, University of Bern, Bern, Switzerland; 10 Practice Mental Health Company, Zürich, Switzerland; 11 Public Health Services Amsterdam(GGD Amsterdam), Amsterdam the Netherlands; 12 Department of Public and Occupational Health, University Medical Center, Amsterdam, the Netherlands; 13 South London and Maudsley NHS Foundation Trust, London, UK; 14 Public Mental Health Implementation Centre, Royal College of Psychiatrists, London, UK; 15 Department of Psychiatry and Mental Health, University of Cape Town, Cape Town, South Africa; 16 Great Yarmouth Acute Service, Northgate Hospital/Norfolk & Suffolk NHS Foundation Trust, Great Yarmouth, UK; 17 Department of Psychiatry and Psychotherapy, LMU University Hospital, Ludwig-Maximilians-University of Munich, Munich, Germany; 18 DZPG (German Center for Mental Health), Partner Site München/Augsburg, LMU Munich, Germany; 19 Collaborative Antwerp Psychiatric Research Institute (CAPRI), University of Antwerp, Antwerp, Belgium

**Keywords:** mental health, social determinants, urbanicity, urban planning, pollution

## Abstract

**Background:**

Urbanization, the shift of a growing population into urban areas, is shaping global development across infrastructure, health, and sustainability. Although it brings economic growth, innovation, and improved access to services, it may also impact mental health.

**Methods:**

The present article was prepared on behalf of the European Psychiatric Association and explores the complexity of associations between urbanization and mental health, highlighting both potential risks and opportunities for improvement.

**Results:**

Urban growth often leads to increased population density, social fragmentation, and environmental stressors, including noise, pollution, and reduced green spaces, all of which might account for worsening mental health. Urban residents might be at risk of various mental disorders due to these stressors, accompanied by the risk of social disconnection. Moreover, socioeconomic disparities in urban settings can lead to unequal healthcare access, further contributing to these challenges. However, urbanization also offers unique opportunities to improve mental health through better resource allocation, innovative healthcare solutions, and community-building initiatives. Indeed, cities might serve as areas for mental health promotion by integrating mental health services into primary care, utilizing digital health technologies, and fostering environments that promote social interactions and well-being. Urban planning that prioritizes green spaces, safe housing, and accessible public transportation holds the potential to mitigate some risks related to urban living.

**Conclusions:**

While urbanization presents significant challenges to mental health, it also provides grounds for transformative interventions. Addressing the mental health needs of urban populations requires a multifaceted approach that includes policy reform, community engagement, and sustainable urban planning.

## Introduction

Urbanization can be defined as the process by which rural areas transform into urban centers. It is now mostly observed in low- and middle-income countries (LMIC) and might be posited as a defining characteristic of global development over the past century. This phenomenon carries far-reaching implications for mental health, economic growth, social dynamics, and environmental sustainability. As the world continues to urbanize, with projections indicating that nearly 68% of the global population will reside in urban areas by 2050 [1], understanding the impact of urbanization is crucial for clinicians, researchers, policymakers, urban planners, and caregivers.

From the economic perspective, urbanization is perceived as the process contributing to increased productivity and innovation. Indeed, urban areas are characterized by high economic activity, offering opportunities for employment, entrepreneurship, and technological advancement. The concentration of resources in urban areas can lead to economies of scale and agglomeration benefits, which are vital for economic development [2]. However, the economic benefits of urbanization do not show a uniform distribution, and disparities between urban and rural areas, as well as within urban centers themselves, can contribute to inequalities [3]. Socially, urbanization exerts profound effects on societal structures and cultural dynamics. Urban areas tend to show increasing diversity, bringing together people from various backgrounds and promoting cultural exchange. This diversity can foster the development of vibrant communities and innovative cultural expressions [4]. However, rapid urbanization can also negatively impact social services, lead to inadequate housing, and contribute to the development of informal settlements, which might limit social cohesion and worsen the quality of life. Finally, urbanization presents both challenges and opportunities with respect to environmental sustainability. On one hand, the concentration of population and industry in urban areas can lead to significant environmental degradation, including air and water pollution, waste management issues, and increased greenhouse gas emissions [5]. On the other hand, urban areas have the potential to drive sustainable development through efficient resource use, public transportation systems, and green infrastructure [6].

**Figure 1.**
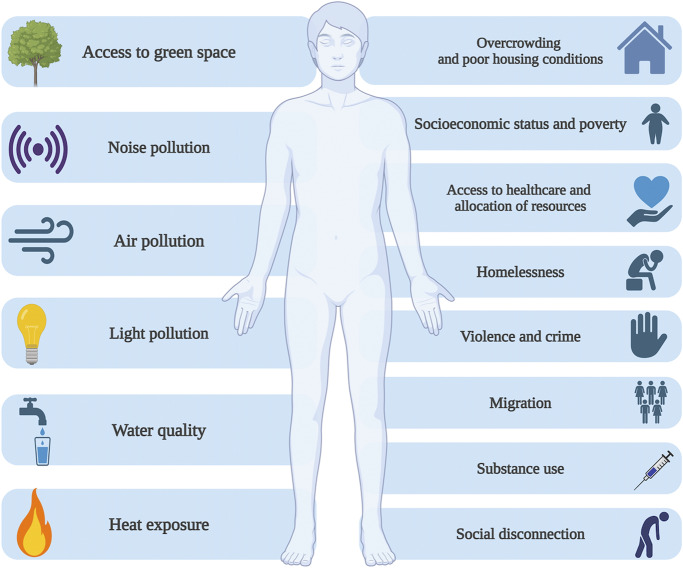
Brief overview of mechanisms underlying potentially detrimental effects of urbanization on mental health outcomes.

All of these multifaceted processes associated with urbanization might also affect mental health through a variety of risk factors that might include: (1) overcrowding and poor housing conditions; (2) negative effects on socioeconomic status; (3) disparities in access to healthcare; (4) increased violence and crime rates; (5) substance use; (6) migration; (7) social disconnection; (8) noise, air, and light pollution; (9) heat exposure, and (10) limited access to green space (Figure 1). The European Psychiatric Association (EPA) recognizes that urbanization is an ongoing process affecting European countries, thus its impact and potential interventions mitigating associated risks require a thorough and balanced discussion. Therefore, the EPA decided to prepare the present position paper (see Supplementary Material for methods), a foundational document to guide the collaboration of EPA with existing European networks, including the City Science Initiative (CSI EU), and to contribute to shaping the urban mental health policy agenda at the European level. Specifically, this position paper aims to articulate the EPA stance on the mental health implications of urban living by (1) summarizing existing evidence in the field; (2) outlining policy areas where action is warranted, (3) providing guidance for future research priorities and public mental health strategies, and (4) contributing to the development of informed, evidence-based urban planning, and clinical practice.

## Mental health in urban and rural areas

A recent analysis of data from 191 countries demonstrated a non-linear association between urbanization and a higher prevalence of common mental disorders [7]. However, for some disorders, including intellectual disability, emotional disorders with onset in childhood, hyperkinetic disorder, and substance use disorders this evidence may not be consistent [8, 9]. Similarly, it remains largely unclear whether the urban–rural gradient is associated with suicide risk as some studies have shown a higher suicide rate in rural areas compared to urban areas, while others have demonstrated the opposite, and some studies have reported no significant differences [10]. These inconsistencies likely reflect methodological variability, differing definitions of urbanicity, and context-dependent sociocultural and economic factors, such as access to care, stigma, and means of suicide.

Differences across specific studies are attributable to difficulties in defining urbanicity. Studies investigating the effects of urbanicity on the prevalence and incidence of mental disorders have conceptualized the exposure using a variety of approaches, such as grouping geographical areas from the most rural to the most urban according to population density, the level of urbanization, density of postal areas, and a dichotomized rural–urban approach. It is also important to consider that the urban environment evolves rapidly, and the results are influenced by the culture of the country where data are collected, making the interpretation even more complex. Specifically, various moderators across different geographic areas need to be considered. For instance, a complex pattern of associations has been observed for major depression. A recent meta-analysis demonstrated that a rural–urban gradient in the prevalence of major depression can be observed in high-income countries (HIC), but not for the general population of LMIC, older adults, and child or adolescent samples [11]. Similarly, it has been shown that the association of urban environment with the risk of non-affective psychosis has been widely replicated across HIC, but not LMIC. Interestingly, the association of urban environment has not been documented for affective psychosis [12]. However, a lack of consistent effect in studies based on samples from LMIC might originate from methodological limitations (e.g., questionable definition of urban exposure, small sample sizes, absences in statistical analysis, and a lack of appropriate regression models) [12].

Causal associations are usually interpreted in light of two hypotheses. The first one, known as the drift hypothesis, posits that individuals with mental disorders move from rural to urban areas because of a greater availability of mental healthcare services and a lower level of stigma in urban areas [13]. The second one – the breeder hypothesis assumes that urban areas are characterized by various risk factors including social disparities, economic hardship, higher levels of violence and crime, and environment pollution [14]. Although both scenarios might be relevant, in the case of some mental disorders, e.g., non-affective psychosis, the association has also been observed for urban birth and upbringing [15]. Moreover, it has been reported that individuals at clinical high risk of psychosis and those with first-episode psychosis show a similar level of neighborhood-level exposures (i.e., proportion of single-parent households, ethnic diversity, and multiple deprivation) that is significantly higher compared to healthy controls [16]. These observations contradict the drift hypothesis.

## Potential mechanisms

### Overcrowding and poor housing conditions

Direct consequences of urbanization are related to increased population density posing a risk of overcrowding that appears when the number of inhabitants exceeds the capacity of available living space. A limited living space has been widely associated with an increased risk of disease transmission and noise exposure thereby contributing to the occurrence of physical health impairments [17]. However, it is also important to note potential consequences for mental health and psychological wellbeing. These consequences might originate from a lack of privacy and negative impact on interactions between household members [18]. To date, several studies have investigated the impact of overcrowding on mental health characteristics. Some studies have found that overcrowding is associated with higher levels of depressive symptoms [19] or subclinical psychopathology in general [20], whereas other studies have failed to find significant associations [21].

Apart from the impact of overcrowding, it is also important to discuss the effects of housing conditions on mental health. A recent systematic review identified four key areas by which housing might impact health outcomes in general including neighborhood or context (e.g., safety issues and access to green spaces), physical building (i.e., all physical aspects of housing), housing market (i.e., financial aspects of housing), and housing policy (i.e., health impact of home ownership versus being a tenant and house stability) [18]. Another systematic review that specifically focused on mental health characteristics (i.e., depressive and anxiety symptoms, psychological impairment, allostatic load, mental strain, and psychological health) revealed a negative impact of mortgage delinquency, housing mobility, a lack of housing tenure, subjective perceptions of inadequate housing, eviction, and poor physical housing conditions [22]. Importantly, the authors found that these associations are also significant across longitudinal studies.

### Socioeconomic status and poverty

Socioeconomic status is a multifaceted concept, encompassing various factors such as financial standing, occupation, education, and living conditions, all of which are closely linked to mental health [23]. Socioeconomic disadvantage has been shown to have a detrimental impact on mental health, contributing to a wide range of mental disorders [23]. This effect is often shaped by both individual socioeconomic deprivation and the broader community context, highlighting the importance of not only the “‘microenvironment,” but also the “macroenvironment” [24].

Socioeconomic stratification leads to disparities in access to essential resources, which play a crucial role in helping individuals mitigate exposure to the “microenvironment” harmful stressors. Higher income allows people to secure critical factors that support mental well-being, such as food security [25], stable housing [26], and better access to professional mental healthcare [27]. Conversely, stress related to poverty may not only contribute to the emergence of depressive and anxiety symptoms, but it may also lead to their worsening over time [28]. It has been shown that changes in income are closely linked to the prevalence of mental health disorders, with those consistently living in poverty experiencing the highest rates of these conditions, followed by individuals who moved in and out of poverty, and finally, those who were never poor, who had the lowest prevalence [29]. Additional socioeconomic constraints, including income instability, perceived job insecurity, and debt accumulation, are also closely associated with poor mental health [30].

Nevertheless, studies examining individual and neighborhood-level socioeconomic factors among native citizens suggest that the broader neighborhood environment – the “macroenvironment,” has a stronger influence on mental health than individual characteristics – the ‘microenvironment’ [31]. Neighborhood poverty might be linked to poorer mental health outcomes beyond the influence of age, education, and income of the individuals [31].

For decades, it has been well-established that socioeconomic status significantly influences the occurrence of mental disorders, particularly in urban areas [32]. Individuals residing in socially disadvantaged and impoverished urban areas experience higher rates of mental disorders, such as depression, anxiety disorders, psychosis, and suicide risk, compared to those living in affluent neighborhoods [33–36]. This phenomenon can be especially observed in economically disadvantaged neighborhoods of high-income areas or HIC [23]. This evidence forms the foundation of the “income inequality hypothesis,” which suggests that socioeconomic disparities contribute to worsening mental health [37]. On the other hand, a number of studies supports the “mixed neighborhoods hypothesis.” It suggests that the presence of higher-income individuals in a neighborhood leads to overall improvements in living standards, access to resources, and mental health [37].

Moreover, exposure to socioeconomic disadvantage during childhood has particularly harmful effects on mental health. Children growing up in poverty are significantly more likely to experience poor mental health later in life compared to their peers from more advantaged backgrounds [38]. The risk increases with both the duration and severity of the disadvantage. This connection is evident across various mental health conditions, including ADHD [39], schizophrenia [40], and depression [41], and is consistent across different regions, from HIC to LMIC. This impact persists regardless of parental mental health or education levels [40]. As similar to findings in adults, there is now strong evidence highlighting the impact of neighborhood socioeconomic conditions on the mental health of young people. Those born and raised in urban and socially disadvantaged areas of HIC are at a heightened risk of developing non-affective psychotic disorders [42].

There are many sociological and biological explanations for the influence of the socioeconomic state on mental health, also in urban areas. Socioeconomic factors including food insecurity, financial stress, and education appear to have a more consistent impact on mental health risks in LMIC than income and employment [41]. Lower education levels are linked to poorer mental health and higher suicide risk, as education affects social status and income [43]. Families with limited resources often struggle to meet basic needs, increasing the risk of mental disorders and chronic stress, which can affect parenting and children’s opportunities. Moreover, recent neuroimaging studies show that lower neighborhood socioeconomic status affects the dysregulation of certain neuronal pathways in the brain of children and adolescents, increasing their mental health risk [44].

Poor mental health can also affect earnings and increase financial stress, creating a bidirectional relationship between socioeconomic disadvantage and mental health [45]. This phenomenon contradicts the theories proposed to explain the observed association between depression and poverty, namely “social causation” and “social drift.” [46]. Factors including education and income are causally linked to mental disorders, while mental disorders can also affect socioeconomic factors [47]. Understanding these interactions, by rigorously planned studies, is essential for developing effective prevention strategies and identifying key intervention targets.

### Access to healthcare and allocation of resources

Access to healthcare is a fundamental element of effective healthcare systems worldwide [48]. However, only a minority of people with mental disorder globally receive treatment with far less coverage in low- and middle-income countries [49].

Significant differences in access to healthcare between rural and urban areas are also observed. In rural areas, reluctance to seek healthcare often stems from cultural and financial barriers. The situation is exacerbated by the lack of available services, a shortage of qualified health professionals, inadequate public transportation, and limited Internet access. Additionally, it is more challenging in these regions to attract and retain healthcare professionals, as well as to provide healthcare services at a level comparable to that of urban areas [50]. On the other hand, urbanization poses distinct challenges in the realm of mental health. This underscores the need for a more comprehensive understanding of the accessibility of mental health services in urban settings to effectively enhance health policy planning [51].

One of the key aspects of access to specialized psychiatric care is location. A study conducted in the US [52] found that residents of more urbanized counties have an easier access to mental health services, with travel times of less than 3 minutes to the nearest facilities. In contrast, some residents of rural areas in the state had to travel over an hour to reach the nearest mental health facility, which posed an additional challenge due to a lack of resources or transportation options [53].

Interestingly, it has also been shown that the presence of mental health services does not guarantee that patients will seek them out sooner. In the study investigating delays in accessing psychiatric treatment for patients with psychosis, no differences were observed between rural and urban areas [54]. Despite proximity to psychiatric centers, higher education, and higher incomes, urban patients did not begin utilizing psychiatric care significantly earlier than those from rural areas [55].

### Violence, crime, and homelessness

From a global perspective, crime and violence tend to be more pronounced in certain urban areas and this phenomenon might be exacerbated by urbanization. Previous studies have shown significant associations among crime, health, and well-being [56]. Crime in a given area is a significant factor influencing mental health, with important implications for preventive measures and health policy. Effective interventions, tailored to local needs, that focus on the causes of crime and its prevention, as well as the allocation of services to areas with high crime rates, can improve mental health of communities [57].

It has been suggested that neighborhood crime can affect mental health both directly and indirectly. Experiencing or witnessing a crime in the community increases the risk of developing mental disorders [58]. However, there is less evidence and consensus regarding whether living in communities with higher levels of crime and violence exerts clear impacts on mental health outcomes, and what the underlying mechanisms [56, 57]. Crime in the neighborhood can act as an environmental stressor, triggering stress responses in areas perceived as dangerous or leading individuals to avoid certain activities. Since this indirect impact of crime can affect entire communities, understanding whether and how neighborhood crime influences mental health could be crucial for public health interventions [57].

The problem of homelessness is another important phenomenon, frequently overlooked due to the expansion of urban areas [59]. The development of cities exacerbates isolation of homeless individuals, making it harder for them to access employment, healthcare, and support services, all of which are essential in addressing homelessness [60]. Previous studies have indicated that homelessness has a negative impact on mental health. Homeless individuals are more likely to experience depression, suicidal thoughts, and substance use disorder (SUD). Qualitative research also highlights the emotional consequences of homelessness, as these individuals not only have to cope with harsh weather conditions but also with stigmatization and persecution, which worsen their health status [61]. Moreover, treatment non-adherence remains one of the main causes of poor health outcomes among individuals experiencing homelessness [62].

A meta-analysis on suicidal behaviors among homeless individuals revealed that aggressive behaviors, mood and psychotic disorders, and addictions are significantly associated with suicide-related outcomes in this population [63]. Additionally, the prevalence of dementia among homeless individuals might be significantly higher than in the general population [64]. Dementia may contribute to the cycle of homelessness, where housing instability increases the risk of cognitive decline, and cognitive impairments make it progressively harder to resolve homelessness [65].

### Substance use

The connection among urbanization, mental health, and substance use is multifaceted. Possible connections include a rise in the supply of both legal and illegal substances, influenced by economic growth and globalization. In urban areas, the growing demand for substances may be a response to lifestyle changes, evolving social and cultural norms, and the environmental stresses that appear in parallel with living [53]. A study conducted in urban and rural areas of Poland showed that daily tobacco smoking is more common in cities than in rural areas [66].

Homelessness is another factor that connects SUD and urbanization. A study in New York, which evaluated homeless individuals entering the emergency room of a city hospital, showed that these patients had higher rates of excessive alcohol consumption over the past year, as well as use of drugs such as heroin, prescription opioids, and a history of opioid overdoses [67]. In a study on homeless adolescents, it was shown that they are more prone to use substances like cocaine, methamphetamine, and heroin compared to their non-homeless peers [55]. The overrepresentation of racial, ethnic, and sexual minorities in the homeless teen population may be indicative of ongoing systemic racism and homophobia [55].

A study involving adolescents explored the link between urbanization and gender differences in smoking, binge drinking, and cannabis use. The results showed that substance use among girls rose as urbanization increased, while boys’ substance use remained constant [68]. In another study comparing the risk factors for substance use in urban and rural areas, it was found that living in an urban environment increases the risk of SUD among girls. The authors proposed that social pressure, the easy access to psychoactive substances, and the cultural norms in large cities may play a role in this increased risk [69].

### Migration

Migration, which involves relocating a person’s main place of residence, whether within a country or across borders, plays a key role in the growth of urban areas. Current migration patterns are primarily influenced by economic opportunities in cities, as well as factors that drive people away from rural regions [70]. More than 60% of the global refugee population resides in urban areas, with this percentage likely being even greater in countries of the Global North [71]. Epidemiological studies have shown that refugees and forcibly displaced individuals are at risk of developing anxiety, PTSD, psychotic disorders, and depression [72, 73]. Furthermore, postmigration stress related to living in shelters under precarious conditions, bureaucratic barriers, reduced access to the healthcare system or weather are key risk factors for mental disorders [74].

Research indicates that mental health services for refugees should consider gender-specific needs and the evolving nature of these needs. It is essential to address economic improvements, alongside social benefits, throughout the resettlement process for both genders. As integration progresses, efforts should focus on reducing social disconnection, with special attention to alleviating the stress of life adaptations [75]. Studies show that social networks of migrants in cities play a key role in improving mental health [76]. The impact of migration-related factors on migrants’ mental health shows that factors such as traveling on foot and educational background are associated with higher levels of anxiety, while pregnancy is linked to an increased risk of depression [73, 77]. Research also indicates that during the COVID-19 pandemic, migrants were particularly vulnerable to harsh living conditions, including inadequate hygiene standards, which heightened the risk of infection. Additionally, limited access to urban healthcare facilities has been found to further worsen their physical and mental health [78, 79].

Besides international migration, the movement from rural to urban areas is also a key factor. While there is conflicting evidence on the mental health of migrants compared to non-migrants, there is a strong support for the idea that social exclusion and social defeat negatively affect the mental health of migrants. The most critical factors might include limited access to labor rights and experiences of stigmatization, discrimination, racism, and inequality [80]. Moreover, children of rural–urban migrants are more likely to experience unfavorable mental health outcomes than their urban counterparts, while also having restricted access to public services [81].

Of note, some phenomena that might be of importance for planning the development of urban areas have been found to moderate the effects of ethnic minority status on mental health. It has been suggested that a greater ethnic density might be protective with respect to the risk of mental disorders and its symptoms. Recent meta-analyses have found protective effects of a greater ethnic density for psychotic experiences, the risk of psychosis, and suicidal ideation [82, 83]. However, evidence for other health outcomes, including common mental disorders, represented by depression and anxiety disorders, is less robust [82].

### Social disconnection

Social disconnection includes two phenomena, i.e., social isolation and loneliness. The first one is objective and informs that the number of social connections is lower than average. In turn, loneliness is the perception that the quality and/or quantity of social bonds is insufficient. Although both constructs seem to be similar, they are modestly correlated and thus might show different underlying mechanisms [84]. A recent meta-analysis revealed that the prevalence of loneliness in Europe ranges between 1.8 and 24.2% depending on age and regional variations [85]. Indeed, the highest prevalence rates of loneliness were found in older adults and those residing in eastern European countries. However, these findings originate from studies performed before the COVID-19 pandemic, and little is known about post-pandemic estimates [86]. Social disconnection, especially loneliness, shows bidirectional associations with mental health [87, 88].

Urban areas offer geographical proximity between people, yet this may not translate into meaningful social connections. In general, cities offer more social opportunities; however, the transient nature of urban populations can make it difficult to form lasting relationships. Difficulties in developing lasting social connections in urban areas may also appear as a consequence of high anonymity, social fragmentation (i.e., due to high diversity of populations living in urban areas), and a greater engagement of urban inhabitants in virtual connections. However, studies comparing the level of social disconnection between urban and rural areas have not provided conclusive findings [89–91]. A potential explanation underlying these discrepancies might originate from the fact that urbanization is a dynamic process and thus investigating the prevalence of loneliness across the rural–urban dichotomy may not consistently uncover clear differences. Therefore, more robust associations might be uncovered by the focus on processes associated with urbanization. For instance, a population-based study in Germany did not reveal significant differences in the level of loneliness between rural and urban areas [92]. However, the authors noticed that some processes, at least theoretically associated with urbanization, might be related to loneliness. These associations were found for regional social transformations (i.e., relocation, emigration, and immigration) and the level of remoteness. Moreover, while much attention has been given to social disconnection in urban contexts, the experience of loneliness in rural areas may be underestimated or oversimplified. Rural communities are often portrayed as socially cohesive, yet they may face unique risk factors for social isolation, including geographic dispersion, outmigration of younger populations, aging demographics, and limited access to social infrastructure and mental health services. These structural challenges, compounded by potential stigma around mental health, may hinder social integration and help-seeking.

### Pollutions

The growing prevalence of noise, air, light, and water pollution in urban environments has emerged as a significant factor influencing mental health, contributing to a range of psychological disorders and exacerbating existing conditions among city dwellers [93]. Independent of hearing ability [94], major effects of noise pollution include disruptions to circadian rhythms, which contribute to the development and progression of chronic non-communicable diseases, such as cardiovascular disease [95], metabolic disorders [96], cancer [97], and respiratory issues [98]. According to the European Environment Agency (EEA), exposure to noise poses a significant threat to public health, impacting not only physical but also mental well-being [93]. Epidemiological studies provide strong evidence linking noise exposure to unfavorable mental health outcomes, including depression, anxiety, behavioral problems, and suicide. Road traffic noise is associated with a 4% increase in depression risk and a 12% rise in anxiety odds per 10 dB(A) [99]. Aircraft and railway noise also contribute, with greater risks from combined exposures [100].

Air pollution has emerged as another significant environmental factor influencing urban mental health [101]. In 2022, 96% of people living in urban areas of the EU were exposed to fine particulate matter (PM) levels that exceeded the health standards recommended by the WHO [102]. Pollutants, including PM2.5 and PM10, ozone (O_3_), nitrogen dioxide (NO_2_), sulfur dioxide (SO_2_), and carbon monoxide (CO), have been linked to negative health outcomes [103]. SO_2_, NO_2_, and PM2.5 have been associated with higher levels of anxiety symptoms, with some studies indicating that long-term exposure may increase the risk of anxiety and related mental disorders [104, 105]. Vulnerable populations, including pregnant women and children, appear to be especially susceptible, with evidence suggesting that early exposure to air pollution can contribute to anxiety and developmental impairments [106, 107]. Studies across different countries focusing on the effect of ambient air pollution on depression have produced mixed findings. It has been shown that exposure to pollutants, including PM2.5, NO_2_, and O_3_ can lead to neuroinflammation and oxidative stress, which are associated with a higher risk of depression [108]. The relationship between air pollution and depression varies, with some studies indicating strong links between long-term exposure and increased risk [108], while other studies suggest that short-term exposure may also play a role [109]. Moreover, a large study using data from the UK Biobank demonstrated an association between air pollution and affective symptoms, which was mediated by differences in the volume of brain structures involved in reward processing and moderated by genes related to stress response [110]. The link between exposure to air pollution, specifically PM2.5, NO, and NO2, and an elevated risk of developing dementia has also been found [111]. Recent studies indicate that air pollution may have significant effects on children and adolescents. Exposure to poor air quality in early-life years may be linked to a higher risk of depression, psychosis, personality disorders, and suicide-related outcomes [112, 113]. Previous studies have also associated air pollution with an increased risk of autism spectrum disorder (ASD), focusing on various exposure periods, such as during pregnancy and early childhood [114]. Evidence highlights that prenatal exposure to pollutants, especially PM2.5, may increase the risk of ASD [115]. Some studies have also found that exposure to other pollutants, including NO_2_ and PM10, also plays a role, though the findings show some heterogeneity [116].

Light pollution, caused by excessive artificial lighting, is an intensively researched urban pollution that increasingly affects human health and the environment. It impacts about 80% of the global population and has been linked to various health outcomes, including neurodegenerative diseases such as Alzheimer’s disease (AD) [117]. Disruptions to sleep and circadian rhythms from light pollution, particularly caused by dim artificial light at night (dLAN), may contribute to the onset of AD by impairing melatonin production and contributing to a neurotoxic protein clearance in the brain [118]. Additionally, light pollution can exacerbate mood disorders and ASD [119, 120]. It has been shown that exposure to dLAN may also be associated with increased symptoms of depression, anxiety, and sleep disturbances [121].

Water pollution is a significant concern, particularly in low-income communities, where exposure to contaminants exacerbates health risks [122]. Previous studies have suggested that heavy metals pose the greatest risk due to their accumulation in the body, leading to increased risk of ADHD and ASD (lead, cadmium), as well as depression (arsenic) [123–125]. While pharmaceutical contaminants are found in low concentrations, their long-term neurological effects remain uncertain [126]. Environmental disasters, such as oil spills and water crises, may further impact mental health. Studies following the Deepwater Horizon and Exxon Valdez spills, as well as the Flint, Michigan water crisis, show increased rates of PTSD, anxiety, and substance use, with lasting effects [127]. Similarly, floods, being one of the most frequent natural disasters, are associated with increased risk of poor mental health outcomes. Survivors often experience prolonged anxiety, stress, flashbacks, and depression, with PTSD rates reaching about 15% across studies [128]. The severity of mental health outcomes is influenced by various factors represented by resource scarcity, personal loss, and the level of exposure [129]. In urban areas, water contamination and flood-related crises disproportionately impact marginalized communities, intensifying existing mental health disparities [130].

### Access to green and blue spaces

The association between exposure to green and blue spaces (GBS) and urban mental health is increasingly being investigated. In the EEA member countries, green infrastructure, encompassing green and blue areas like allotments, private gardens, parks, street trees, water bodies, and wetlands, accounts for an average of 42% of urban spaces [131]. Exposure to GBS typically refers to the frequency with which individuals interact with or access these environments, though it can also involve one-time interventions [132]. Most studies have indicated that green spaces consistently contribute to health improvements across both urban and rural areas [133]. Exposure to GBS has been linked to general mental health benefits in all age groups [134, 135]. These benefits include reducing the risk and alleviating the symptoms of mood disorders, psychosis, and developmental disorders [136].

It has been revealed that there is a strong connection between greenspace exposure and lower rates of depression [137] and its symptoms [138]. While some studies have found a significant relationship between greenspace and reduced anxiety symptoms, these findings are inconsistent [139]. However, a large study based on neuroimaging, environmental, and genetic data demonstrated that greenness might be negatively correlated with an anxiety symptom group, and this relationship was found to be mediated by brain regions involved in emotion regulation and moderated by variation in the *EXD3* gene [110]. Exposure to green spaces has also been associated with a reduced risk of schizophrenia [140], with some evidence supporting a dose–response relationship [141]. It has been indicated that greater greenspace exposure in early childhood may be associated with a lower risk of psychosis in adulthood, except for schizoaffective disorder [142]. According to a study conducted on a small sample, blue spaces may aid in managing symptoms of racing thoughts associated with mania and rumination linked to depression [143].

Access to green space has also been associated with fewer hospital admissions because of schizophrenia spectrum disorders [144]. Research on the impact of access to green space on children’s mental disturbances has revealed mixed results. Green space exposure is inversely related to the risk of attention-deficit/hyperactivity disorder (ADHD) [145]. Additionally, it has been associated with reduced symptoms of inattention and hyperactivity in clinical and non-clinical populations [146], although findings are inconsistent for healthy children [147]. Green space exposure has been linked with less severe internalizing and externalizing behaviors in the general population [148], but the evidence varies and may be influenced by sociodemographic factors [149]. For instance, children from socioeconomically disadvantaged backgrounds may derive greater psychological benefit from access to green areas, potentially due to higher baseline stress or fewer alternative resources. Conversely, in neighborhoods characterized by a population of low socioeconomic status, the quality, safety, or accessibility of green spaces may be limited, attenuating their potential impact.

### Heat exposure

Global temperatures show an increasing trend over recent years and it has been predicted that this process will continue together with an increase in the frequency of heat waves by the mid-21^st^ century [150]. These observations are the consequence of widely acknowledged climate changes [151]. A global warming process also appears to be facilitated by ongoing urbanization [152]. The impact of high ambient temperatures has been extensively documented for physical health impairments. However, less is known about the consequences for mental health. A recent systematic review demonstrated that high ambient temperatures might affect mental health [153]. The authors revealed consistent associations of this exposure with suicide risk. Moreover, they observed associations with increased risks of mental health-related admissions and emergency department appointments. Findings with respect to other mental health outcomes were not consistent (i.e., mood disorders, schizophrenia, organic mental disorders, dementia, alcohol and substance use disorders). High temperatures have also been associated with increased rates of violent and aggressive behaviors [154]. There are also studies showing negative consequences of heat exposure for cognitive and educational performance [155, 156].

Several mechanisms might underlie the effects of heat exposure on mental health [157]. First, it is needed to note that high ambient temperatures might lead to sleep disturbance. Experimental studies have also demonstrated that high temperatures at night lead to sleep disturbances that manifest in reduced slow-wave and rapid eye movement sleep, sleep interruptions, and increased body movements [158, 159]. Second, high temperatures might alter central thermoregulation, impact neurotransmission, increase the blood–brain barrier permeability, and affect the brain blood flow [157].

## Discussion

### Summarizing the evidence

The present position paper identified a number of processes that might explain associations between urbanicity and poor mental health outcomes (Figure 1). However, evidence from longitudinal studies still appears to be scarce, thereby limiting insights into potentially causal mechanisms. There is also a heterogeneity across definitions of urbanicity that might limit the potential to generalize specific findings. Moreover, little is still known on how urbanization affects vulnerable populations, e.g., individuals with disabilities, LGBTQ+ populations, and refugees. These groups often face unique challenges in urban environments, such as increased exposure to discrimination, social disconnection, and limited access to tailored mental health services. For example, persons with disabilities may encounter physical and infrastructural barriers that restrict their participation in community life. Similarly, LGBTQ+ individuals and refugees may experience heightened stress due to stigma, marginalization, and difficulties integrating into urban social networks, all of which can impact mental health. Another limitation is that the focus on single mechanisms does not inform about interactive effects of processes associated with urbanization. The rationale behind the implementation of more complex analytical models to study the effects of urbanicity on mental health originates from the fact that processes related to urban living can act both as risk and protective factors. Moreover, their influence occurs at different time points. Additionally, these factors affect each other, creating feedback loops [7]. Thus, complexity arises not only from the multifactorial nature of the systems but also from the dynamic interactions between these factors. These interactions not only vary in intensity and nature but also undergo changes over time. A multifactorial landscape and temporal dynamics of processes behind urbanization may underlie a non-linear association of urbanicity with common mental disorders [7].

### Approaching data complexity

As a foundation for meaningful policy and intervention efforts, research should begin with the collection of detailed, interdisciplinary epidemiological data [160]. Subsequent studies should explore the biopsychosocial and economic factors underlying urban mental health challenges, paving the way for contextually appropriate and culturally informed solutions. Studying urban and rural mental health requires complex scientific approaches with analytical methods that can integrate the large number of influencing factors and the underlying data. In the more traditional scientific approaches, symptoms are often studied using analytical techniques based on linear regression, applied to data from a single point in time. Innovative network analyses of long-term and time series can provide new insights into intra-individual symptom dynamics in relation to contextual variables. Complexity science focuses on ecosystems that are adaptive and operate via feedback loops and circular relationships. Solutions therefore, are often collective (rather than individual) and focus on multiplicity and interdisciplinarity.

Network models might be of particular importance for better understanding of temporal patterns between multiple processes behind urbanization that might affect mental health and wellbeing [161]. Network approaches conceptualize psychopathology as a range of dynamic interactions between specific symptoms and underlying mechanisms that ultimately result in the emergence of specific manifestations of psychopathology. Importantly, network modeling might be implemented in the analysis of data collected using a variety of designs, including cross-sectional and longitudinal data, without the use of a predefined model of causal inferences. Longitudinal data, especially those intensively collected across several timepoints, e.g., using the experience sampling method (ESM) [162], might hold a particular promise for understanding temporal ordering of processes determining mental health in urban areas by providing insights into a range of contextual factors. Network models of ESM data might be useful in dissecting the phenomena that show potentially causal associations (by means of analyzing temporal networks) from those that show momentary co-occurrence patterns (by means of analyzing contemporaneous networks) [163]. Finally, by the analysis of centrality metrics that illustrate the importance of specific variables, network models may guide the development of specific interventions.

### Informing the interventions

Effective public mental health interventions exist to prevent and treat mental disorders, limit associated impacts [164], and promote mental wellbeing and resilience, including in urban settings. Interventions to address risk factors are important to both prevent mental disorders but also prevent relapse in those with existing mental disorders. Different types of intervention might be provided by different sectors. However, high-risk groups require more targeted approaches to prevent the widening of inequalities. Only a minority of people with mental disorders receive treatment, far fewer receive interventions to prevent associated impacts, with negligible coverage of interventions to prevent mental disorders. In particular, new urban migrants will require dedicated support, especially considering that the proportion of the global population living in urban areas is projected to rise to at least 68% by 2050, primarily in developing and emerging economies [160]. The implementation gap results in population-scale preventable suffering, broad impacts, and associated economic costs. There is an opportunity to assess the size of the implementation gap in urban settings with public health and then set out the required actions to address the gap through coordinated action between sectors. An important step is to assess the coverage of various evidence-based interventions, including those for high-risk groups. This can be done in collaboration with public health bodies. These activities might further inform opportunities to address the gaps through coordinated approaches by different sectors.

To date, little is known about an optimal set of interventions that need to be implemented while planning the development of urban areas. A recent analysis of data from a sequential survey of a multidisciplinary group of researchers, practitioners, advocates, and young people revealed key priorities that might serve as actionable targets to develop cities as mental health-friendly areas [165]. These priorities cover life skills needed for personal development, valuing and accepting ideas and choices, providing safe public space for social contacts, employment and job security, centring the input of young people in urban planning and design, and addressing adverse social determinants. These priorities were analyzed across six levels of factors related to urbanicity, i.e., personal, interpersonal, community, organization, policy, and environment. Identified priorities indicate that young people center their needs around personal growth, opportunities for building social connections, and inclusiveness in decision-making processes. However, it is also important to note their focus on the detrimental effects of social determinants represented by community and household violence. As noted by the authors of this study, there were several interindividual differences across age groups of participants, indicating a clear need for broad inclusiveness while planning the development of urban areas. This observation is also in agreement with findings from a recent study showing beneficial mental health outcomes of engaging the community in the construction of attractive urban places [166].

**Figure 2.**
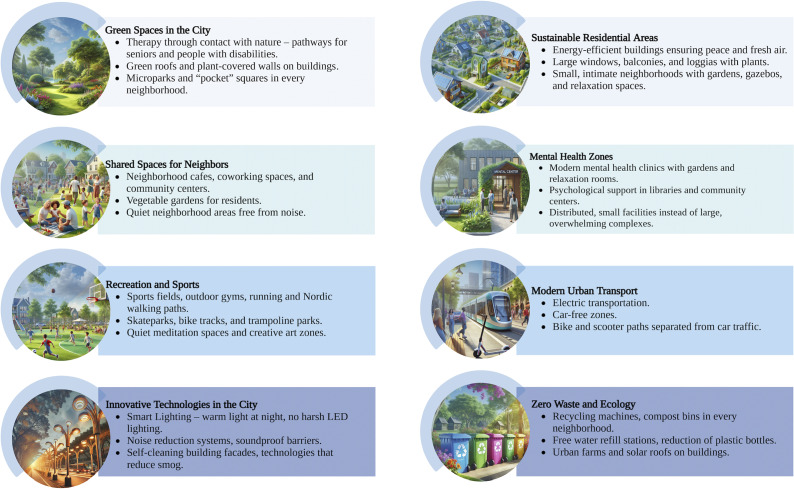
Overview of interventions proposed to improve mental health while developing urban areas.

Although specific risks behind the development of urban areas for mental health have been identified, the efficacy of various interventions remains largely unknown. Based on the findings discussed in the present study, some activities mitigating the risks driven by urbanization might be proposed (Figure 2). To date, some studies have investigated whether interventions focused on these targets might improve mental health. For instance, a recent simulation study showed that a successful implementation of an extensive street greening strategy in Barcelona might result in significant improvement of mental health outcomes, such as poor self-reported mental health, visits to mental health specialists, the use of antidepressants and tranquilizers or sedatives. These improvements would correspond with annual savings of over 45 M EUR [167]. There are also initial signals that improvements in housing conditions might result in beneficial effects on mental health and quality of life [168].

It is further important to highlight that urban areas offer several opportunities for the development of various strategies promoting mental health. For instance, digital media has the potential to provide significant adaptive benefits as well, particularly in urban environments, where its accessibility fosters supportive online communities, facilitates identity exploration, and offers emotional relief, especially for marginalized groups [169]. In this context, AI emerges as a transformative tool in mental health, offering innovative approaches to awareness, diagnosis, intervention, and support [170]. The AI-driven solutions, such as machine learning, natural language processing, and digital phenotyping, facilitate early detection of mental disorders, personalized treatment plans, and real-time monitoring through wearable devices and chatbots [170, 171]. These technologies hold promise in addressing the growing mental health burden in urban environments, where stressors such as social isolation, noise pollution, and fast-paced lifestyles contribute to psychological distress. Recent studies highlight the ability of AI to enhance mental healthcare by providing scalable and cost-effective solutions, particularly chatbot-supported applications, as well as other AI-driven virtual therapists and teletherapy platforms [172]. Randomized controlled clinical trials and systematic reviews have shown that the use of chatbots is associated with significant reductions in depressive symptoms, generalized anxiety, and psychological distress, with effect sizes ranging from moderate to large [173–175]. Additional benefits may include improvements in sleep disturbances, reductions in perceived loneliness, and attenuation of stress-related and disordered eating symptoms, depending on chatbot design and users’ behaviors [173, 176, 177]. Moreover, AI supports clinicians by analyzing large datasets, identifying patterns in mental health disorders, and predicting responses to treatment [178]. However, ethical challenges remain, including concerns about data privacy, algorithmic bias, and the need for culturally sensitive AI models [179].

In light of these findings, several concrete policy measures may be considered by European cities and governments. These could include: setting minimum green space accessibility standards (e.g., by ensuring all residents live within a 300-meter radius of publicly accessible greenery), implementing urban noise reduction strategies (e.g., noise barriers, traffic calming zones, and green buffers), integrating mental health considerations into urban and transport planning (e.g., quiet routes, public transport designed to reduce crowding, and expanding cycling infrastructure), and supporting socially inclusive housing policies that reduce segregation and improve living conditions. In addition, particular emphasis should also be placed on the increasing influx of new urban migrants, who represent a vulnerable population for whom mental health and socioeconomic support systems remain largely underdeveloped or inaccessible. To address this gap, policymakers might consider the establishment of community-based psychosocial support hubs in high-density migrant neighborhoods, offering culturally sensitive mental health services, employment counseling, and integration support in collaboration with local stakeholders. Moreover, policy frameworks may incorporate youth-led participatory planning, the development of safe and inclusive public spaces, and educational programs that support life skills, agency, and resilience. They should include measures and initiatives aimed at promoting youth employment and job security, as well as addressing adverse social determinants, including community and household violence. Furthermore, policymakers should actively support the implementation of digital technologies, including AI-driven tools. This may include the development and regulation of evidence-based digital mental health platforms — such as chatbots, virtual therapy, or early detection systems — to ensure equitable access, cultural sensitivity, and data privacy. While the policy measures outlined above highlight key domains for innovation, comprehensive urban mental health strategies should be grounded in a systematic consideration of the full spectrum of risk and protective factors, their socioecological contexts, and the dynamic interactions between individual-, community-, and policy-level determinants.

Currently, many European urban areas are implementing initiatives aimed at improving the mental health of their residents through city-based actions [180–182]. Examples of such initiatives include Helsinki, where integrated service chains and easily accessible psychological support for children and youth are being developed, providing free, confidential, and holistic care. In Rotterdam, the “Green Connection” project creates walking routes that connect communities with green spaces, promoting physical activity and mental wellbeing. Meanwhile, Stockholm uses the Individual Placement and Support method, offering people with long-term mental health problems the assistance of a job coach, which enables their successful integration into the labor market. These examples demonstrate that cities can effectively support mental health through actions integrated with urban planning, healthcare systems, and local social policies.

Although many risk factors for mental health in the social and physical environment have already been identified, there is a need for more in-depth investigation. It is particularly important to consider the cumulative impact of multiple simultaneous exposures and their interactions, especially in relation to different population groups such as children, older adults, or people experiencing homelessness. Research is needed to more precisely determine how specific factors contribute to the development of specific mental disorders. Another important direction is the search for novel, previously underexplored risk factors that may gain importance in changing social and environmental conditions, such as the digitalization of daily life or housing crises.

### Promoting collaborative efforts

The EPA recognizes the challenges we will face in the upcoming years in the context of urbanization and its effect on the population, especially the most vulnerable individuals – our patients. As psychiatrists united in the EPA, we can make a substantial contribution by shaping the European agenda for urban mental health together. The forementioned need is the reason to look for networks that can reinforce the vision of the EPA. In line with this, the question is how the EPA can connect with one of the networks that enriches Europe, namely the European City Science Initiative (CSI EU), by creating a working group.

“CSI EU aims to strengthen the ways in which science and research can help address the urban challenges and to develop a structured approach to evidence-informed policy making at cities level” (https://openresearch.amsterdam). It is a European self-organizing, informal network that is constantly looking for connection and collaboration between cities, universities, and politicians. “It is aiming to work on better societies to battle for a better future by working together in all layers of society, to accelerate the transition to inclusive, resilient, safe, climate-proof and resource-efficient eco systems” [183]. The CSI EU network includes various EU organizations represented by, i.e., UNIC (the Network of Universities from Capitals of Europe), EUROCITIES (the network of major European cities), EUKN (the European Union Knowledge Network), 100 Resilient Cities, and Covenant of Majors [183]. With respect to the UNICA, several research groups are involved. These include clinical and research units representing various specialties, including psychiatry, sociology, architecture, and machine learning. Also, the European Commission representatives are part of the network being intensely involved and supporting this initiative wholeheartedly [183]. In June 2019, stakeholders decided in Amsterdam (the Netherlands) to focus the CSI EU on five specific urban challenges with the involvement of specific cities: air quality (Paris, France), circular economy (Hamburg, Germany), mental health (Thessaloniki, Greece), sustainable urban mobility (Cluj-Napoca, Romania), and tech and the city (Reggio Emilia, Italy) [183].

### Concluding remarks

In sum, the present position paper provides information about a range of potential risks related to urbanization that might affect mental health. However, it is also needed to note that urbanization may serve as a process that occurs in parallel with the implementation of strategies that improve mental health and create a more supportive and resilient environment for all residents. Before designing rigorous studies that will focus on developing specific interventions and polices, it is still needed to better understand how specific factors related to urban growth interact across time and space. In this regard, future studies addressing the impact of processes behind urbanization need to adopt approaches focused on the collection of real-life data representing a variety of contextual factors, environmental exposures, and mental and physical health characteristics.
